# Aberrant Diagnostic Imaging Resulting in Misdiagnosed Acute Perforated Appendicitis: A Case Report

**DOI:** 10.7759/cureus.54304

**Published:** 2024-02-16

**Authors:** Christian Przeslawski, Leanne Iorio, Jeffrey Gerken

**Affiliations:** 1 General Surgery, Corewell Health Southeast - Farmington Hills Campus, Farmington Hills, USA

**Keywords:** intraabdominal appendix locations, appendicitis misdiagnosed as diverticulitis, ct evaluation acute appendicitis, diagnostic laparoscopy in perforated viscus, diagnostic laparoscopy in acute abdomen

## Abstract

A 31-year-old male with a history of diverticulitis presented for acute abdominal pain and was found to have several small areas of free air on computed tomography (CT) of the abdomen/pelvis. Due to inflammatory changes seen around the sigmoid colon and small bowel, he was diagnosed with perforated diverticulitis. The patient complained of significant right-sided abdominal pain with significant tenderness on abdominal examination. The patient was initially treated with diagnostic laparoscopy and was actually found to have acute perforated appendicitis with mild appendiceal adherence to the sigmoid colon. This case highlights the importance of careful history and physical examination in an era where imaging often precedes the surgeon's evaluation. The case also provides support for laparoscopy in select cases of pneumoperitoneum, sparing patients the morbidity of undergoing an open laparotomy.

## Introduction

Acute appendicitis is one of the most common surgical issues, with 300,000 appendectomies being performed every year in the United States and a lifetime risk in the United States of approximately 9%. Despite the commonality of the diagnosis and treatment, acute appendicitis frequently presents with aberrant clinical presentations [1,2]. We present a case in which initial diagnostic imaging was consistent with perforated sigmoid diverticulitis; however, the definitive diagnosis at the time of surgery was acute perforated appendicitis.

## Case presentation

A 31-year-old male presented to the emergency room at a community hospital with two days of severe, constant, suprapubic, and right-sided abdominal pain that began suddenly. Due to worsening pain and difficulty with ambulation, he presented to the hospital. He also had chills, anorexia, nausea without emesis, and felt feverish. He complained of three weeks of constipation prior to the onset of the abdominal pain. Three months earlier, he had similar symptoms and was diagnosed with diverticulitis, which was treated with oral antibiotics. He was seen at a different hospital without available electronic records for our viewing. He was recommended to have a colonoscopy at that time but had not yet had one performed. Other than asthma, the patient had no other chronic health issues. He denied a personal or known family history of colonic malignancy or inflammatory bowel disease. He denied prior abdominal surgical procedures.

**Figure 1:**
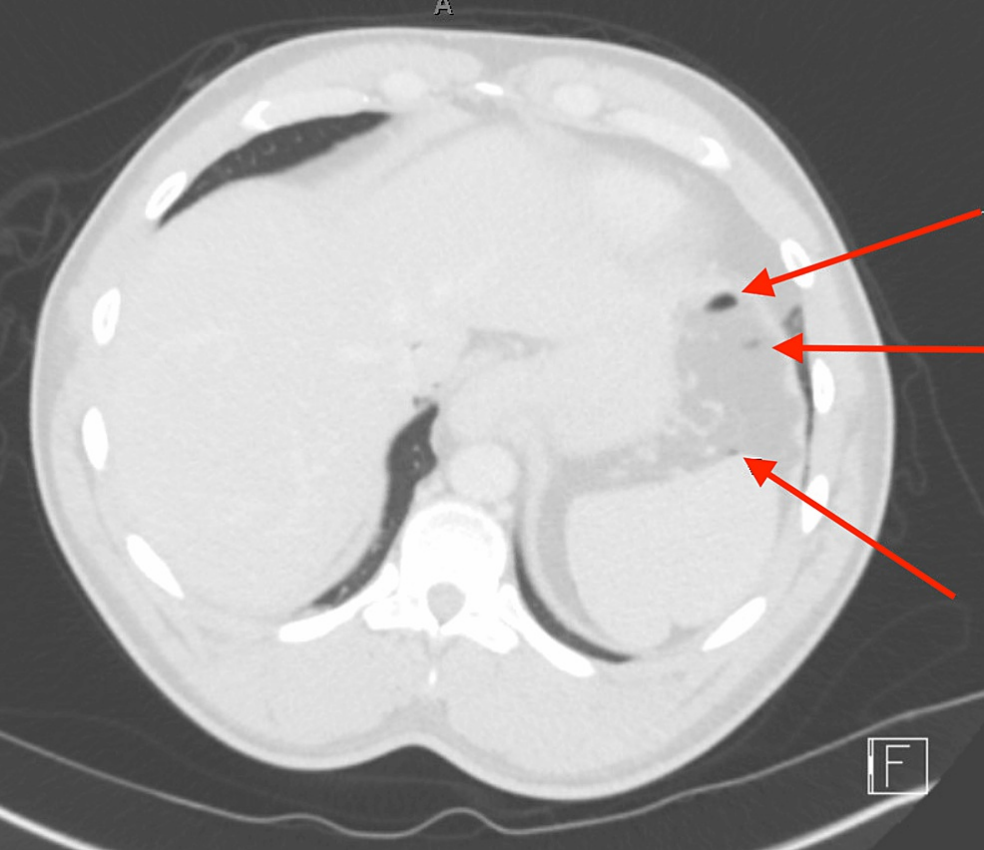
Axial CT image showing punctate air within the abdominal compartment.

Upon arrival, he was evaluated by the emergency medicine team and was noted to be afebrile and hemodynamically stable. Computed tomography (CT) of the abdomen/pelvis with intravenous (IV) contrast was performed with board-certified radiologic interpretation of inflammation of the sigmoid colonic wall and diverticula consistent with diverticulitis, possible small bowel fistula to the sigmoid colon, small bowel dilation, and evidence of pneumoperitoneum (Figure 1). At this point, our surgical team was called, and the patient was examined. He appeared uncomfortable and diaphoretic with a distended abdomen with tenderness to palpation and involuntary guarding in the suprapubic area and right lower quadrant. He also exhibited rebound tenderness in all four quadrants. Significant laboratory values were a leukocytosis of 13,000 with 11,400 neutrophils. After our evaluation, the patient was resuscitated with IV fluids, started on IV antibiotics, and offered a diagnostic laparoscopy due to the small foci of free air on the CT scan.

**Figure 2:**
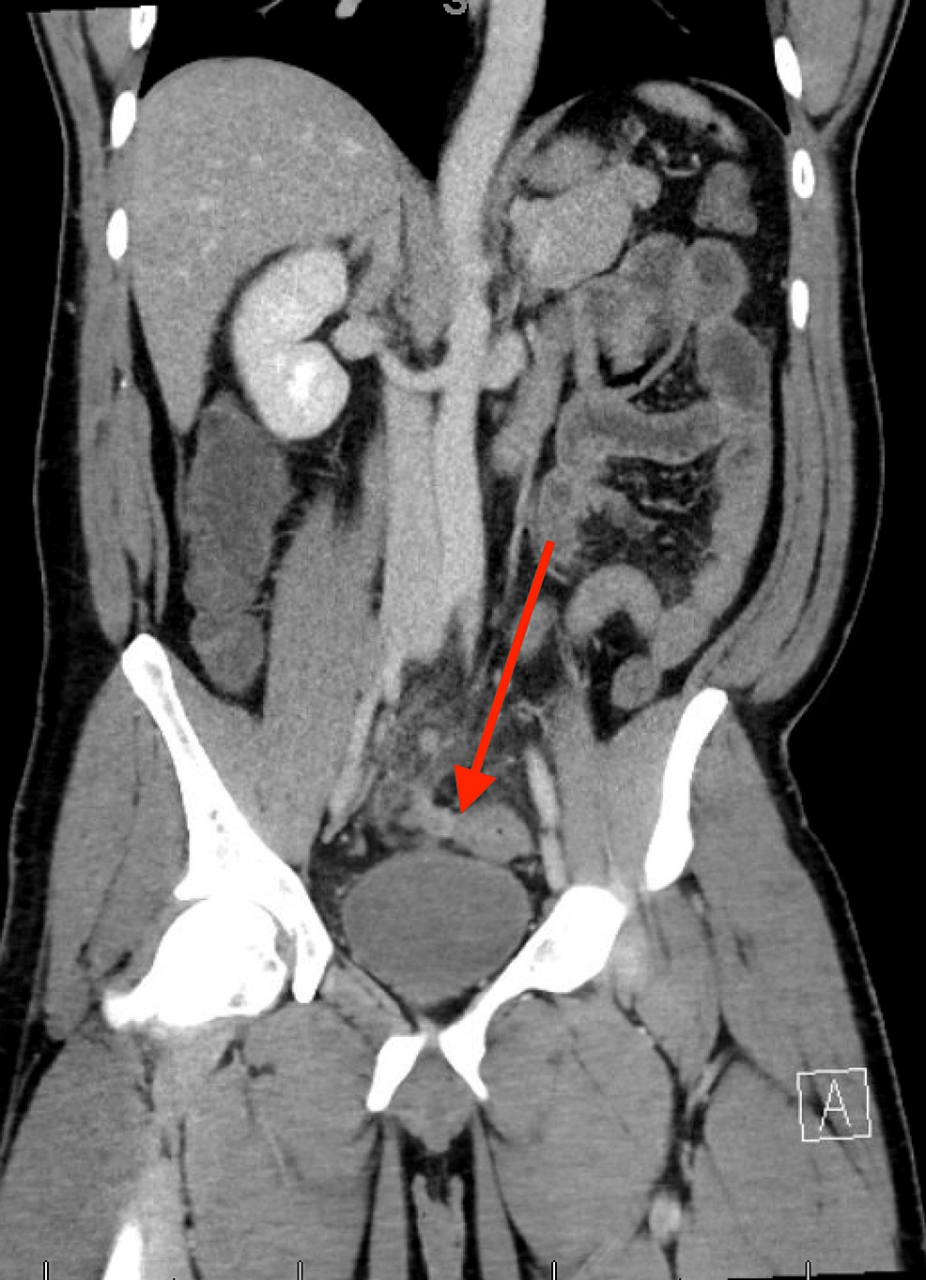
Coronal image of the CT on admission showing the close approximation of the appendix with the sigmoid colon.

Upon entry to the abdomen with the laparoscope, a phlegmon and interloop abscesses were seen surrounding the terminal ileum. The rest of the abdomen was inspected and noted to have diffuse inflammation and purulence extending up to the liver without evidence of feculent contamination. The bowel was then carefully run with the release of the interloop abscesses starting at the terminal ileum. The small bowel dived into the pelvis and was adherent to the sigmoid mesentery. The small bowel was gently peeled away from the sigmoid mesentery without appreciation of any small bowel perforation. The appendix was then identified and was stuck to the sigmoid colon with a perforated tip. The appendix was dissected off the sigmoid colon, and the appendiceal base was stapled across using an endoscopic gastrointestinal anastomosis stapler. The sigmoid colon appeared intact. The abdomen was then thoroughly irrigated, and two drains were placed, one in the pelvis and the other in Morrison’s pouch. His postoperative course was uneventful, with his drains removed, and his hospital discharge occurring on postoperative day four. Further retrospective review of the CT showed close approximation of the appendix to the sigmoid colon (Figure 2). Follow-up in the office two weeks after surgery showed an uneventful healing course. He is scheduled to obtain a CT abdomen/pelvis and colonoscopy outpatient.

## Discussion

Reliance on imaging findings can occasionally obscure the clinical picture. In this case, the history and physical examination may have aided in the diagnosis of acute appendicitis. The patient initially complained of predominantly right-sided abdominal pain. He did endorse nausea and constipation, which have been described multiple times as common symptoms of appendicitis. "Cameron's Current Surgical Therapies" describes the "classic appendicitis" presentation as dull periumbilical pain that progresses to focal pain in the right lower quadrant and is often associated with nausea and emesis. However, this is only true in about half the cases of acute appendicitis [1]. In this case, the CT scan had been ordered prior to the surgical team's examination of the patient, which raises the question of when a CT scan should be ordered in possible acute appendicitis cases. Recently, there has been increased demand for CT imaging. Naoum et al. encourage the use of CT imaging as it decreases the rate of misdiagnosis for acute appendicitis from 26% to 6% [3]. Kabir et al. performed a review in 2017 of the different diagnostic tests available for diagnosing acute appendicitis, including lab values, imaging modalities, and diagnostic scoring systems. Their overall conclusion was that low-dose CT scans should be considered for diagnosing acute appendicitis due to sensitivity similar to repeat ultrasound and high-dose CT scans [4]. The review by Kabir et al. did not provide specifics as to when to order CT scans. Tan et al. proposed an algorithm using the Alvarado Score to determine which patients would benefit most from CT scans [5]. The original Alvarado Score was created by Alfredo Alvarado in 1986, measuring several signs, symptoms, and lab values listed in Table 1. This score was simply a diagnostic predictor of appendicitis, and an algorithm was not proposed for the next step in a patient’s care [6]. Tan et al. recommend further imaging in males scoring 4 to 6 and women scoring 4 to 8 on the Alvarado score while recommending diagnostic laparoscopy for males with a score of greater than 7 and women greater than 9 [5]. Performing a thorough history and physical in conjunction with laboratory values allows one to calculate an Alvarado score which can guide management. In this patient’s case, his score would be 8, and a CT scan might not have been necessary. The patient could have theoretically avoided the time undergoing a CT scan and proceeded directly with diagnostic laparoscopy.

**Table 1:** Signs and symptoms when calculating the Alvarado Scoring System [6].

Alvarado Scoring System	Value
Symptoms	
Migration of pain	1
Anorexia	1
Nausea/emesis	1
Signs	
Tenderness in the right lower quadrant	2
Rebound tenderness	1
Elevation of temperature >37.3 °C	1
Laboratory values	
Leukocytosis	2
Left shift	1

An additional important consideration in this case is the aberrant clinical presentation of patients, in addition to the aberrant anatomical position where the appendix may lay in the abdomen. This patient did not have an initial period of transient periumbilical abdominal pain evolving to a right-sided focus seen in “classic” appendicitis. As stated above, nearly half of the patients with appendicitis do not present classically. It is also essential to point out the different locations where the appendix can be located. Per Sibileau et al., pelvic appendicitis can be difficult to differentiate diagnostically from sigmoid diverticulitis [7]. Cameron describes the positions and the frequency of those locations as retrocecal 64%, subcecal 32%, pelvic 2%, preileal 1%, and postileal 0.5% of cases [1]. Mesocoeliac appendicitis has been described in the literature as well. Sibileau et al. note that this presentation occurs when the appendix is positioned between multiple bowel loops in the periumbilical region and presents as a febrile occlusion [7]. This patient had an appendix that was located within loops of the small bowel and was in contact with the sigmoid colonic wall in the pelvis, resulting in reactive sigmoidal colonic inflammation. It is important to note that the patient could have had a mild case of appendicitis when he had presented to an emergency department two months prior to our encounter with the patient. Perhaps imaging findings of inflammation in the pelvis obscured his diagnosis at that time, resulting in the diagnosis of acute diverticulitis.

A final point in this case is that mild to moderate perforation in a hemodynamically stable patient can be approached safely laparoscopically. Navez and Navez published a 2013 literature review regarding the safety of the laparoscopic approach to the acute abdomen. They note that relative contraindications to laparoscopic approaches are hemodynamic instability, feculent peritonitis, massive abdominal distension, and perforated cancers. They go on to cite decreased abdominal wall morbidity, as well as overall morbidity with laparoscopic appendectomy versus open appendectomy. They do note that further research is needed in the case of perforated diverticular disease [8]. In this case, the diagnostic laparoscopy yielded perforated appendicitis rather than diverticular disease and spared the patient the morbidity of an open laparotomy. In select cases, a perforated viscus can be safely treated with laparoscopy in hemodynamically stable patients exhibiting small-volume pneumoperitoneum without feculent peritonitis.

## Conclusions

This case underscores the critical importance of a thorough and comprehensive history and physical examination in an era of increasing access to diagnostic imaging. The Alvarado Score can be utilized to aid in clinical decision-making regarding acute appendicitis and determining when further imaging should be pursued. Additionally, this case highlights a less common positioning of the appendix in the deep pelvis, resulting in reactive sigmoid colonic inflammation that appeared similar radiographically to sigmoid diverticulitis. Although imaging is extremely helpful in aiding diagnosis and surgical planning, it should not replace a comprehensive history and physical exam. Finally, this case provides support that select cases of pneumoperitoneum can be approached safely laparoscopically to spare patients the morbidity associated with a laparotomy in select situations.
